# EHR Sampling Interval Bias Detection and Burden of Blood Pressure Excursions: Implications for Clinical Decision Support and Model Validity in Pediatric ECMO

**DOI:** 10.3390/info17020135

**Published:** 2026-02-01

**Authors:** Neel Shah, Ethan Sanford, David R. Busch, Ranveer Singh, Saurabh Mathur, Jayesh Sharma, Philip Reeder, Sriraam Natarajan, Lakshmi Raman

**Affiliations:** 1 Department of Pediatrics, Washington University in St. Louis, 660 S. Euclid Ave., St. Louis, MO 63130, USA; 2 Department of Pediatrics, University of Texas Southwestern Medical Center, Dallas, TX 75390, USA; 3 Department of Anesthesiology and Pain Management, University of Texas Southwestern Medical Center, Dallas, TX 75390, USA; 4 Department of Neurology, University of Texas Southwestern Medical Center, Dallas, TX 75390, USA; 5 Department of Biomedical Engineering, University of Texas Southwestern Medical Center, Dallas, TX 75390, USA; 6 Department of Computer Science, University of Texas at Dallas, Richardson, TX 75080, USA; 7 The Anne Burnett Marion School of Medicine, Texas Christian University, Fort Worth, TX 76104, USA

**Keywords:** electronic health records, extracorporeal membrane oxygenation, blood pressure, pediatrics, hypotension, hypertension

## Abstract

Routine Electronic Health Record (EHR) blood pressure charting under-samples dynamic physiology, risking missed hemodynamic instability. This study quantifies how HER-like down-sampling changes the detection and burden of hypo- and hypertension versus continuous monitoring and articulates the consequences for clinical decision support and machine learning label quality. We retrospectively analyzed 78 ECMO-supported pediatric patients (2019–2023). The continuous mean arterial pressure (MAP) captured every 5 s was resampled at intervals from 5 s to 1 h. We screened for 3 min windows of hypotension or hypertension at 10th/90th age-normed thresholds, comparing the per-patient event frequency and burden with EHR-derived recordings. At 10th/90th thresholds, hypotension events fell from 13,936 (5 s) to 3803 (15 min; −72.7%); the EHR captured 3471. Hypertension events dropped from 1573 to 410 (−73.9%); the EHR registered 1587. The EHR data overstated hypertension burden, indicating preferential documentation during prolonged instability while missing brief excursions. Standard EHR sampling significantly under-reports blood pressure derangements in pediatric ECMO. This underreporting of brief events may limit the accuracy of clinical decision support tools and machine learning algorithms in high-acuity patients. High-frequency data acquisition improves event capture and should be prioritized for clinical decision support and machine learning development.

## 1. Simple Summary

Bedside monitors record blood pressure every few seconds, but Electronic Health Records (EHRs) often include a value only every 15–60 min. We analyzed the continuous mean arterial pressure from 78 children on ECMO (one value every 5 s), counted short low- and high-pressure episodes, and measured the time outside age-appropriate ranges. We then repeated the analyses after down-sampling and compared them with actual EHR entries. Sampling every 15 min caused about three-quarters of brief episodes to vanish. EHR snapshots also overstated the time spent hypertensive, likely because providers chart more during instability. Using higher-frequency vital signs (ideally continuous, at least every 5 min) can better capture true instability, strengthen research, and make decision support safer.

## 2. Background and Significance

Intensive care monitors stream sub-minute vitals, yet informative observation processes in the EHR (irregular, state-dependent documentation) create a sampling bias. When labels for instability are derived from these sparse samples, event misclassification and burden distortion propagate into clinical research, CDS rules, and ML models. Most prior studies rely on sparsely sampled EHR vitals to link abnormal blood pressure with outcomes. However, the detection of significant clinical events has been shown to be underrepresented in the standard EHRs, particularly in critically ill patients [1]. Undetected events jeopardize AI-driven decision support by degrading training data fidelity [2].

Studies comparing high-frequency data to EHR-derived data for key physiological variables, including heart rate, blood pressure, and oxygen saturation, demonstrate significant event detection gaps compared with hourly EHR data [1]. Furthermore, the electronic medical record is prone to an increased risk for errors, further compounding the risks associated with inaccurate measurements [3]. In critical care settings, the bedside availability of high-frequency data offers improved insights into the physiological trajectory of a patient’s clinical state, facilitating the possibility of timely and informed interventions. Keene et al. showed that inconsistent vital sign charting hindered the early recognition of deterioration [4]. High-frequency data platforms mitigate such risks, providing a more accurate representation of dynamic patient states found in critically ill patients.

The adoption of high-frequency data has been most notable in pediatric cardiac intensive care units. One prominent example is the Etiometry (Boston, MA, USA) platform, which captures physiologic and laboratory data at a resolution of one data point every 5 s. This platform has been leveraged to create a hyperlactatemia index [5] to non-invasively predict low cardiac output syndrome [1,6]. Similarly, the inadequate delivery of the oxygen index (IDO2) algorithm uses available physiological and laboratory variables to predict the probability of low mixed venous saturation. Studies have demonstrated the utility of the IDO2 index in predicting cardiac arrest following the surgical repair of congenital heart disease [7]. In another multi-center study, investigators utilized real-time risk algorithms utilizing IDO2 for the successful vs. unsuccessful weaning of vasoactive agents [8]. Furthermore, recent studies have demonstrated that algorithms generated with high-frequency data have been able to help predict the risk of extubation failure in these high-risk patient populations.

Asfari et al. demonstrated that EHR vital sign data underrepresented significant events [5] compared to a high-frequency data platform. Prior failures of machine learning and artificial intelligence tools in the prediction of sepsis and other forms of clinical deterioration [9] may be the result of sampling bias and datasets which lacked sufficient granularity. Bias and the limited generalizability of such artificial intelligence (AI) systems are also well-documented when built on low-resolution data [10].

Patients supported on extracorporeal membrane oxygenation (ECMO) represent one of the sickest cohorts in the intensive care unit, with marked hemodynamic fluctuations, particularly during ECMO initiation. This study aimed to quantify the frequency of hypotensive and hypertensive events across multiple sampling frequencies using high-frequency data and compare these findings to traditional EHR data. This single-center study was conducted with pediatric and cardiac ICU patients supported on ECMO and focused on blood pressure, as it is highly predictive of poor outcomes [11]. These findings would inform clinicians and researchers of the minimal frequency that would capture clinically relevant changes in a critically ill population and provide crucial information on the sampling frequency required to generate next-generation predictive algorithms.

## 3. Materials and Methods

This retrospective single center study utilized archived vital sign data of pediatric patients (0–19 years of age) supported on ECMO. Patients were admitted between November 2019 and June 2023. Patients were selected based on the inclusion and exclusion criteria (Supplementary Materials File S1) of a larger NIH funded study exploring utilizing high-frequency data to predict neurologic injury in this population. The University of Texas Southwestern institutional review board approved the study (STU 2021–0095). Data were collected from a maximum of 24 h prior to ECMO initiation through the entire ECMO course. The final dataset included 78 patients, with data spanning up to 15 days after cannulation onto ECMO.

Blood pressure was the sole vital sign that was analyzed in this study, as it was the most predictive of adverse outcomes. All patients included in this study had continuous invasive blood pressure monitors in place. Continuous 5 s measurements were exported from the Etiometry platform (Boston, MA, USA). Both continuous MAP and EHR MAP values were derived from the invasive arterial blood pressure monitor. The EHR MAP field is auto-populated from the invasive arterial-line monitor with nurse verification; nurses may manually enter MAP values per clinical practice. Median sampling was utilized to prevent the smoothing of values, which may prevent the detection of hypotension or hypertension events.

Data cleaning stages included noise reduction in the mean arterial pressure (MAP) data, for which a three-stage filtering process was applied. First, we excluded values outside the plausible range of 20–180 mmHg. Second, we excluded measurements exhibiting implausible change patterns, defined as transient step-changes >10 units over 5 s that were sustained for less than 300 s. This often occurs when the arterial line is being accessed for lab draws. Finally, we excluded sequences <200 s surrounded by missingness and then forward-filled remaining gaps; we did not impute deleted values.

Recognizing the wide age-based variability in blood pressure, MAP values were age-standardized using inpatient MAP percentiles [1,12]. Hypotension and hypertension were quantified using the following metrics:

Event Frequency: The count of non-overlapping 3 min windows per patient in which every resampled MAP value crossed the threshold; this definition was pre-specified to examine how coarser sampling (fewer points per window) inflates/deflates counts.

Burden: The normalized area under or over the MAP curve for a given threshold, calculated using the trapezoid method and expressed in mmHg-second. We then divide this quantity by the total duration of ECMO in seconds. The resulting burden metric is an average excursion of mmHg normalized by ECMO duration.

The event frequency and burden were chosen to align with previously published work [13].

We consider three threshold values to determine hypotension and hypertension based on age-specific percentiles: below the 5th/above the 95th, below the 10th/above the 90ths, and below the 25th/above the 75th percentiles. Primary endpoint: The difference in per-patient event frequency across sampling intervals vs. continuous 5 s data at 10th/90th percentiles. Secondary endpoints: (i) The difference in burden (mmHg-normalized by ECMO duration) across intervals; (ii) the agreement with EHR-recorded values; (iii) sensitivity analyses at 5th/95th and 25th/75th thresholds [14,15].

To evaluate the effect of sampling frequency and method on the accuracy of event detection, we resampled continuous data utilizing median values across seven sampling frequencies (5 s to 1 h). Lastly, these data were compared to irregularly charted HER-derived values as a measure of routine documentation consistent with current clinical standard practice. Wilcoxon Signed Rank was utilized to compare events and burden to both EHR and 5 s frequency, and a Bland–Altman plot analysis was performed to display data distribution.

## 4. Results

Table 1 summarizes the cohort demographics. The median age of patients was 239.5 days, with 48% being children (1–19 years) and 41% being neonates (<28 days). In total, 51% were male, and the median weight was 8 kg. V-A ECMO was the most common ECMO type—64% of patients—and the majority were placed on ECMO for a respiratory indication. The median length of ECMO duration was 124.1 h, with 85% of patients surviving until hospital discharge.

The quantity of data points over varying sampling frequencies and from the EHR can be seen in Table 2. The median percentage of data that was forward-filled per patient was 0.71%. The median gap length was 30 min prior to forward-fill (IQR 25). The data volume fell from 11,432,379 points at 5 s sampling to 63,717 at 15 min (–99.4%); the EHR contained 29,167 points total (mean 373.9 per patient). The median interval of EHR-sampled data points was 1800 s (30 min), with an IQR of 3180 s.

The median per-patient frequency of hypotension/hypertension windows (10th/90th percentiles) is summarized in Table 3. Hypotension events declined from 12,044 at 5 min to 3803 at 15 min (–68.4%), with the EHR-derived total at 3471. Similarly, mean events declined from 154.4 (5 min) to 48.8 (15 min) per patient. EHR-derived data had a mean of 44.50 hypotensive events noted per patient.

Hypertensive events, defined as events over the 90th percentile for age, similarly had a notable change from 5 to 15 min, with 1573 total events at 5 min but only 410 events found at a sampling frequency of 15 min (Table 3). Similarly, 20.17 mean events per patient were found when sampling at 5 min, but only 5.26 events were found when sampling was reduced to every 15 min. EHR-derived data comparatively had 1587 total hypertensive events, or 20.35 on average per patient. Data when defining hypo- and hypertension at the 5th and 95th percentile, as well as the 25th and 75th percentile, can be found within the supplement (Supplementary Materials, Tables S1 and S2).

Table 3 summarizes statistics about hypotension and hypertension events in 78 patients on ECMO; the total events are the sum across all patients calculated for each patient using the median blood pressure (i.e., median MAP) measurement across different interval sizes from continuous data resampled every 5 s to every hour. For each such frequency, the columns represent the total number of events aggregated over all patients and the mean, the median, and the interquartile range of the number of events per patient in the cohort. EHR-derived data were automatically entered into the medical record per standard practice and verified by nursing staff. Each hypotension (or hypertension) event is defined as a 3 min period where the blood pressure falls below the 10th percentile (or exceeds the 90th percentile) value for the patient’s age group. Inferential statistics completed using the Wilcoxon rank test are shown comparing 5 s and EHR to other sampling intervals.

The overall burden of hypo and hypertension, as defined by the 10th and 90th percentile, is displayed in Table 4. The total hypotension burden was more similar to EHR-derived data, with expected decreases as sampling became more infrequent. Conversely, the hypertensive burden was higher within the EHR-derived data than was found with high-frequency data sampling across all sample rates. Despite the high event counts, the normalized hypotension burden remained modest, indicating that most excursions were short or shallow. Data regarding the burden when defined by the 5th and 95th as well as the 25th and 75th percentile can be found within the supplement (Supplementary Materials, Tables S3 and S4). In addition, we compared the proportion of hypo- and hypertension using the 10–90th percentiles utilizing the 5 s data compared to the EHR. The median proportion of hypotension was 10.4% for the 5 s data, compared to 13.5% for the EHR data. Comparatively, the median proportion of hypertension was 2.2% for the 5 s data and 4.4% for the EHR data.

Table 4 summarizes statistics about the hypotensive and hypertensive burden in 78 patients on ECMO; normalized burdens (average excursion magnitude, mmHg) with higher values indicate more severe and/or more prolonged excursions. The total burden is the sum across all patients, while the mean and median were calculated for each patient using the median blood pressure (i.e., median MAP) measurement across different interval sizes, from continuous data resampled every 5 s to every hour. For each such frequency, the columns represent the total hypotensive and hypertensive burden over all patients and the mean, the median, and the interquartile range of the patient-wise area in the cohort. EHR-derived data were automatically entered into the medical record per standard practice and verified by nursing staff. Hypotensive (or hypertensive) burden is defined as the area of the blood pressure curve (in mmHg-seconds) where its values fall below the 10th percentile (or exceed the 90th percentile) value for the patient’s age group, normalized by the total time in seconds spanned by the observations for that patient. Inferential statistics completed using the Wilcoxon rank test are shown comparing 5 s and EHR to other sampling intervals.

**Figure 1.**
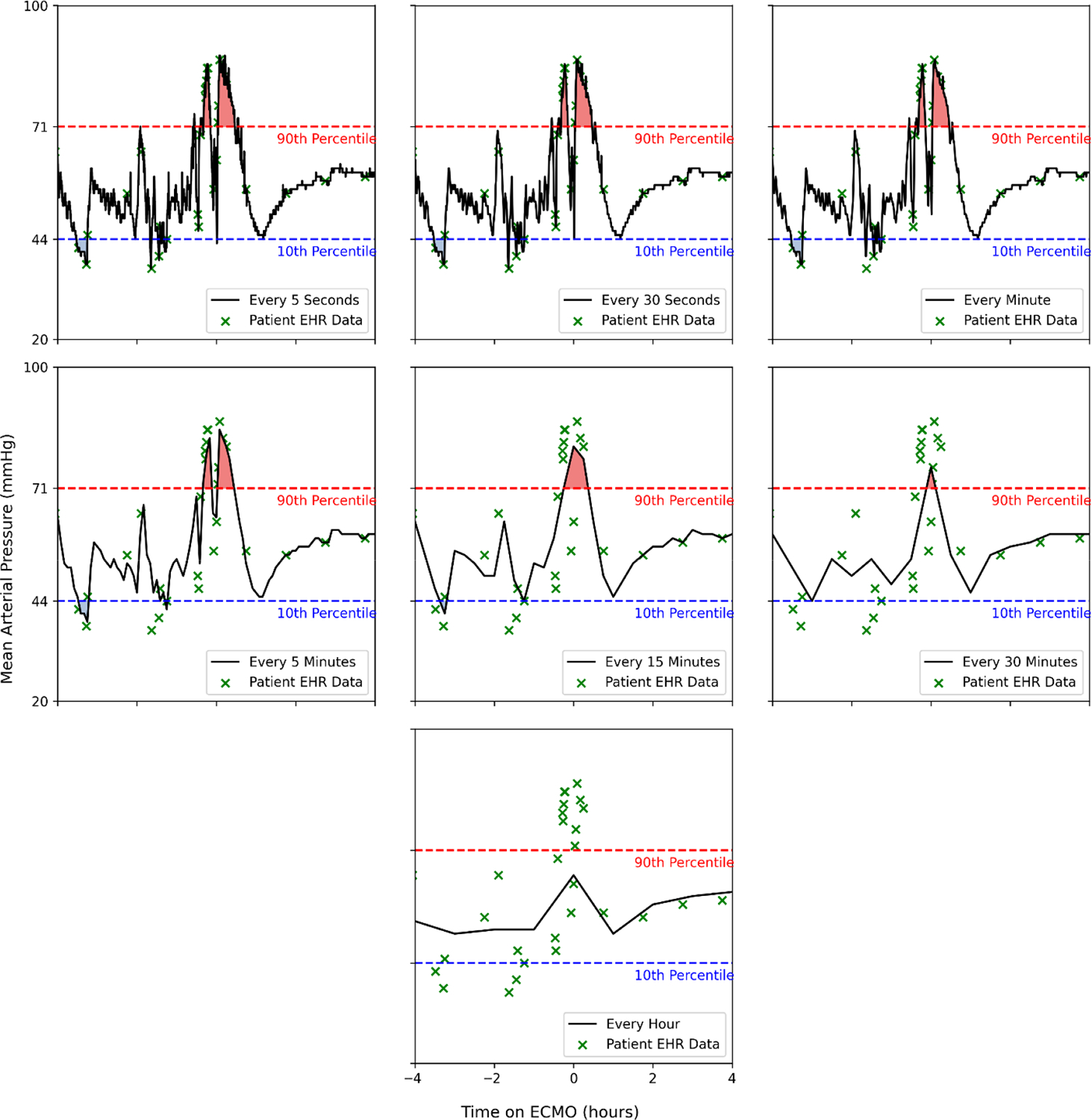
Sampling of a selected patient at every 5 s, every 30 s, every minute, every 5 min, every 15 min, every 30 min, and every hour; the EHR-derived values are seen in green as Xs. The shaded area under the curve represents time spent with hypotension (blue) or hypertension (red) as defined by the 10th and 90th percentiles.

**Figure 2.**
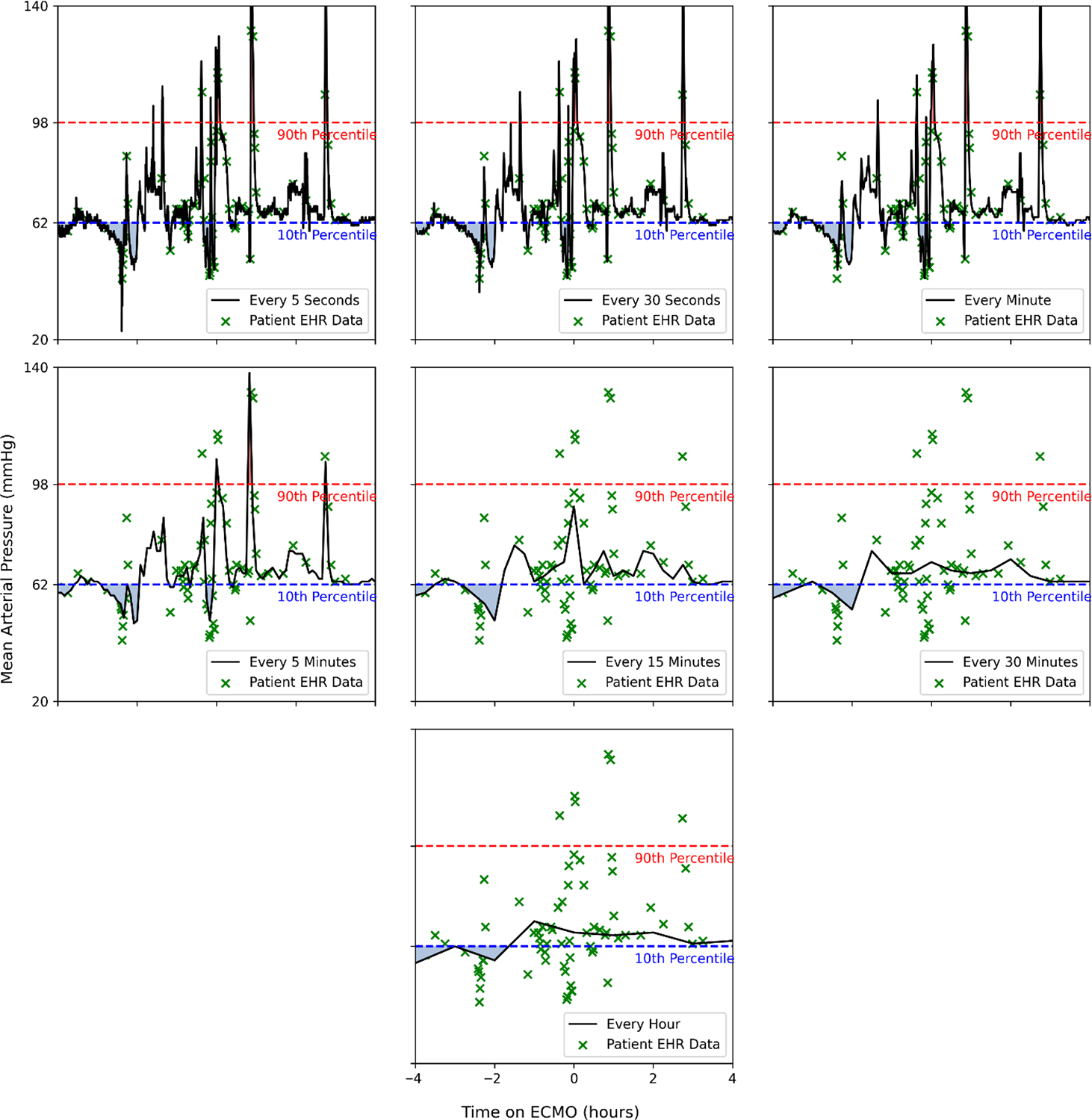
Sampling of a selected patient at every 5 s, every 30 s, every minute, every 5 min, every 15 min, every 30 min, and every hour; the EHR-derived values are seen in green as Xs. The shaded area under the curve represents time spent with hypotension (blue) or hypertension (red) as defined by the 10th and 90th percentiles.

**Figure 3.**
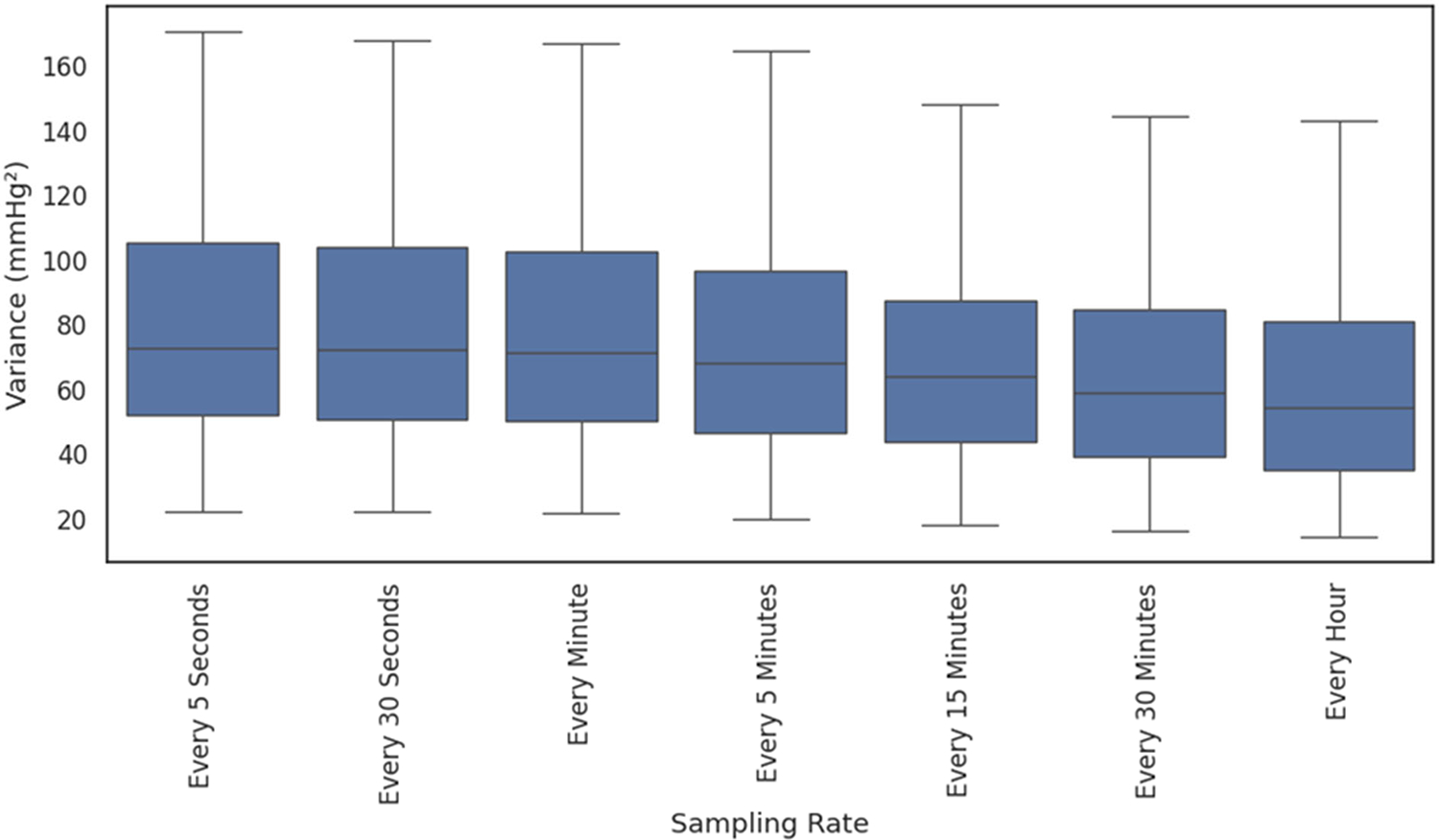
Variance as calculated by the average of squared differences from the mean was calculated for each individual patient and can be seen across varying sampling rates.

Figures 1 and 2 show two illustrative patients; green markers denote EHR-recorded values across sampling strategies. The shaded area under the curve represents the time spent with hypo- or hypertension as defined by the 10th and 90th percentiles. The figures are artificially limited to 240 min pre- and post-ECMO cannulation for ease of comparison. In addition, we calculated the variance for each individual patient across sampling intervals, and this can be seen in a box plot format in Figure 3. To evaluate individual level differences across sampling frequencies and across the EHR-derived sampling frequencies, we have included patient level heatmaps of hypo- and hypertension at the 10/90th percentile within the supplement as Supplementary Materials, Figures S1 and S2. Bland–Altman plots are found within the supplement comparing 5 s and EHR sampling intervals.

## 5. Discussion

This single-center arterial-line study of 78 pediatric ECMO patients demonstrates that observation process bias in EHR charting suppresses brief excursions and inflates burden estimates during prolonged instability. Irregular EHR-derived charting intervals constitute a source of label error: they suppress the detection of brief instability and distort the time-integrated burden. In CDS and ML pipelines, this alters thresholds, inflates false-negatives, and contributes to external validation failures [16]. Paradoxically, detection briefly rises at 30 s and 1 min intervals because fewer points per 3 min window must all breach thresholds. With fewer points per window, the criterion is statistically easier to satisfy, briefly inflating counts before they decline again at longer intervals. As our comparative analyses reveal, the event detection and total burden of abnormal blood pressure defined using the AUC drop sharply at regularly resampled continuous data sample intervals above 15 min [17]. Additionally, standard EHR sampling underestimates the total number of blood pressure excursions. Conversely, the burden of hypo- and hypertension was overestimated in EHR sampling.

The specific reason for EHR sampling overestimating burden compared to 5 s sampling is unknown, but we hypothesize that these results may indicate a bias towards oversampling and EHR reporting of abnormal vital signs during prolonged clinical instability, but a poor ability to detect shorter episodes of clinically significant changes in blood pressure. In addition, as seen in Figure 3, higher-frequency sampling allows for a greater detection of variance from the mean blood pressure in our cohort of pediatric ECMO patients. In addition, inferential analysis showed that EHR data were statistically different at 15 min and higher intervals at capturing hypotension burden, and at all sampling intervals for hypertension burden. These discrepancies highlight that a reliance on sparse data risks masking short-lived but clinically meaningful changes, potentially undermining the deployment of predictive algorithms and AI tools in time-sensitive scenarios while also risking confounding created by the variable sampling of vital signs caused by human input into the EHR [18]. For CDS, excursion rules and burden thresholds should specify a minimum physiologic sampling interval (≤5 min; continuous preferred); when the cadence is sparser, logic should add confirmation windows and display confidence qualifiers, and counts/burdens should be interpreted as lower bounds.

These findings correlate with a broader body of research demonstrating that conventional EHR-based documentation often fails to reflect the magnitude or duration of physiologic shifts in critically ill populations [1]. Such shortcomings have been documented in both adult and pediatric intensive care settings, with transient episodes of hypotension, arrhythmia, or desaturation frequently overlooked. Even with an arterial line streaming beat-to-beat data, retrospective EHR snapshots flatten the signal into sporadic points. Clinically, without this continuity, clinicians and CDSSs alike cannot quantify cumulative hypotensive burden, leaving the kidneys, brain, and myocardium exposed to an unnoticed ischemic debt.

Sparse sampling constrains downstream research and AI/ML development because pivotal vital sign patterns may be missing from training data. The available literature increasingly supports a more granular, continuous-monitoring paradigm to mitigate this critical information gap and improve AI/ML algorithm deployment and reliability at the bedside.

A primary methodological gap is the lack of a consensus on optimal sampling intervals that balance clinical feasibility with data fidelity. Our systematic comparison of intervals from sub-minute to hourly offers a practical guide: each incremental jump in sampling period leads to a disproportionately higher number of missed events, underscoring the fragility of low-resolution data. Our interval comparison demonstrates a steep loss of event detection beyond 5–15 min in this cohort of pediatric ECMO patients. This work builds upon prior work demonstrating the importance of <5 min sampling in other settings [1,9,14]. Closely related is the knowledge gap regarding the role of such brief but consequential episodes in disease trajectories. Historically, hourly or bi-hourly charting intervals were considered sufficient for most clinical documentation, yet our findings suggest that clinically relevant hemodynamic events can emerge and resolve within shorter intervals, necessitating a rethink of standard clinical practice. Additionally, studies seeking to evaluate the relationships between blood pressure and clinical outcomes should ideally utilize the highest frequency input possible, with an emphasis on at least a sampling interval of no greater than 5 min.

For predictive analytics and machine learning models in critical care, high-resolution data can substantially enhance both sensitivity and specificity. Many existing algorithms, particularly those predicting events such as sepsis onset or acute hemodynamic decompensation, are trained on EHR datasets that significantly under-sample key vital signs. Our results indicate that such models may fail external validation or yield inflated false-negative rates simply because essential features—like short-term abnormalities in mean arterial pressure—are absent in the data. By contrast, integrating sub-minute sampling from continuous data aggregation systems unlocks a richer feature space, enabling a more precise detection of early physiologic deterioration. Ultimately, this could translate into timelier interventions and better outcomes. This may explain why several models derived on EHR data have performed poorly when externally validated or when deployed prospectively [10]. Sampling less frequently than every 5 min or simply relying on EHR-derived data, especially in a critically ill population, is highly likely to miss critical events and limit the reliability of any training or validation data.

Despite demonstrating the value of high-frequency sampling, our study has several limitations. First, it was conducted retrospectively at a single center, limiting its direct generalizability across different hospital systems, patient populations, and monitoring technologies. Second, our high-frequency data required rigorous filtering and imputation; errors in raw data from sensor noise or transient disconnects could skew the results of a study utilizing only high-frequency data without filtering, but this could be performed in future work by a machine learning algorithm. Third, we focused on blood pressure as a sentinel vital sign, but other parameters like heart rate variability or oxygen saturation may show different patterns and require tailored sampling strategies. Notwithstanding these caveats, our work offers a valuable roadmap for researchers aiming to refine data collection protocols and analytics pipelines for the next generation of clinical predictive algorithms.

Moving forward, multi-center validation studies are warranted to ascertain whether the threshold frequencies identified here hold true under varied clinical conditions, including a varied acuity of critically ill patients, monitoring modalities, and patient demographics. Additionally, prospective trials that integrate real-time streaming data into automated decision support tools could establish whether more granular sampling demonstrably improves the timely recognition of deteriorations. Future work should also explore advanced modeling techniques such as recurrent neural networks or graph-based learning that inherently account for the temporal structure and complex interactions of continuous physiologic signals. The ultimate goal is to develop adaptive systems that can automatically adjust sampling frequency in high-risk states, achieving a balance between data richness and clinician workflow constraints.

## 6. Conclusions

In this single-center pediatric ECMO cohort, conventional EHR sampling substantially underestimated the frequency and altered the burden of blood pressure excursions. For CDS/ML and bedside analytics, we recommend ≤5 min physiologic sampling (continuous preferred) as a design parameter, understanding that this recommendation is extrapolated from arterial-line MAP and has not been directly validated for other vital signs or populations, and that excursion counts/burden derived from sparser EHR entries should be interpreted as lower-bound estimates unless corrected for the observation process. Conventional EHR sampling intervals consistently underestimate the frequency and severity of hemodynamic instability, limiting the accuracy of patient-centered outcomes researched using both traditional statistical models and AI/ML models. While challenges of implementation remain, ranging from technological integration to data storage, our results affirm that more granular monitoring strategies can yield meaningful gains in fidelity. Closing the gap between real-time physiology and static EHR snapshots is pivotal for safer, more accurate critical care analytics.

## Supplementary Material

information-17-00135-s001

**Supplementary Materials:** The following supporting information can be downloaded at https://www.mdpi.com/article/10.3390/info17020135/s1. Supplementary File S1: Inclusion and exclusion criteria; Table S1: Median hypotension–hypertension events (5–95th percentile); Table S2: Median hypotension–hypertension events (25–75th percentile); Table S3: Median hypotension–hypertension burden (5–95th percentile); Table S4: Median hypotension–hypertension burden (25–75th percentile); Figure S1: Heat Map comparing individual patient level burden of hypotension (10th percentile); Figure S2: Heat Map comparing individual patient level burden of hypertension (90th percentile).

## Figures and Tables

**Table 1.** Characteristics of the study population (*n* = 78).

Age (Days)	239.5 (3–4010)
Age Category, *n* (%)	
Neonates (<28 days)	32 (41%)
Infants (1–12 months)	10 (13%)
Children (1–19 years)	36 (48%)
Male Gender, *n* (%)	40 (51%)
Weight (kg)	8 (3.64–43.8)
ECMO Type VA, *n* (%)	50 (64%)
ECMO Indication	
Respiratory, *n* (%)	45 (58%)
Cardiac, *n* (%)	22 (28%)
ECPR, *n* (%)	11 (14%)
Length of ECMO Run (hours)	124.1 (91.65–230.7)
Survival to Discharge, *n* (%)	66 (85%)

**Table 2.** Quantity of data points over varying resampled continuous sampling frequencies.

	Total Data Points	Mean per Patient	Median per Patient
5 s	11,432,379	146,568.95	109,469
30 s	1,907,360	24,453.3	18,263.5
1 min	954,014	12,230.95	9134
5 min	190,926	2447.77	1827.5
15 min	63,717	816.89	610
30 min	31,893	408.89	305
1 h	15,962	204.64	152.5
EHR-Derived	29,167	373.94	295.5

**Table 3.** Median hypotension–hypertension events (10–90th percentile).

MAP Sample Frequency	Total Events	Mean per Patient	Median per Patient	IQR	Comparison to 5 s (Effect Size and 95% CI)	*p* Value	Comparison to EHR (Effect Size and 95% CI)	*p* Value
Hypotension								
5 s	13,936	178.67	66.00	151.75	Ref	Ref	0.04 (28 to 55)	<0.01
30 s	16,127	206.76	76.00	181.50	0 (−17 to −7)	<0.01	0.03 (36 to 71)	<0.01
1 min	17,634	226.08	80.50	194.75	0 (−27 to −14.5)	<0.01	0.02 (43 to 82)	<0.01
5 min	12,044	154.41	55.50	129.00	0.17 (0 to 11)	<0.01	0.04 (22 to 50)	<0.01
15 min	3803	48.76	17.00	42.00	0 (32 to 62)	<0.01	0.31 (−9 to −2)	0.13
30 min	1814	23.26	8.00	19.50	0 (39 to 74)	<0.01	0.04 (−18.5 to −13)	<0.01
1 h	820	10.51	3.00	9.75	0 (45.5 to 82.5)	<0.01	0 (−29 to −18)	<0.01
EHR-Derived	3471	44.50	27.50	38.00	0.04 (28 to 55)	<0.01	Ref	Ref
Hypertension								
5 s	1525	19.55	19.55	5.50	Ref	Ref	−3 (−4.5 to −1.5)	<0.01
30 s	2005	25.71	25.71	7.00	0 (−3 to −1)	<0.01	0.34 (−3 to 0)	0.55
1 min	2293	29.40	29.40	8.00	0 (−5 to−2)	<0.01	0.29 (−1.5 to 1)	0.33
5 min	1573	20.17	20.17	6.00	0.18 (−1 to 0)	0.08	0.24 (−4.5 to −1)	<0.01
15 min	410	5.26	5.26	1.00	0 (3 to 7.5)	<0.01	0.02 (−13 to −6)	<0.01
30 min	154	1.97	1.97	0.00	0 (3 to 8)	<0.01	0.01 (−14 to −7)	<0.01
1 h	65	0.83	0.83	0.00	0 (3 to 9)	<0.01	0 (−16 to−7)	<0.01
EHR-Derived	1587	20.35	13.00	18.75	−0.22 (−4.5 to −1.5)	<0.01	Ref	Ref

**Table 4.** Median hypotension–hypertension burden (10–90th percentile).

MAP Sample Frequency	Total Burden (mmHg)	Mean per Patient	Median per Patient	IQR	Comparison to 5 s (Effect Size and 95% CI)	*P* Value	Comparison to EHR (Effect Size and 95% CI)	*P* Value
Hypotension								
5 s	36.68	0.47	0.26	0.47	Ref	Ref	0.41 (−42.4 to 18.4)	0.75
30 s	36.46	0.47	0.26	0.46	0.10 (3.9 to 7.0)	<0.01	0.40 (−50.2 to 19)	0.55
1 min	36.34	0.47	0.25	0.46	0.10 (6.9 to 13.9)	<0.01	0.38 (−53.8 to 15.5)	0.49
5 min	35.45	0.45	0.24	0.45	0.04 (31.7 to 61.4)	<0.01	0.32 (−98.1 to −3.4)	0.07
15 min	33.39	0.43	0.22	0.45	0.01 (107.4 to 167.2)	<0.01	0.20 (−192.9 to −48.6)	<0.01
30 min	31.38	0.40	0.19	0.41	0.01 (149 to 271.2)	<0.01	0.13 (−245.4 to −115.4)	<0.01
1 h	28.52	0.37	0.16	0.36	0.01 (276.3 to 449.7)	<0.01	0.11 (−450.1 to −217.3)	<0.01
EHR-Derived	37.32	0.48	0.24	0.41	0.41 (−42.4 to 18.4)	0.75	Ref	Ref
Hypertension								
5 s	18.65	0.24	0.12	0.24	Ref	Ref	0.17 (−433 to −98.5)	<0.01
30 s	18.19	0.23	0.12	0.24	0 (8.3 to 16.9)	<0.01	0.14 (−458.2 to −117.9)	<0.01
1 min	17.86	0.23	0.11	0.24	0 (13.2 to 33.2)	<0.01	0.13 (−469.3 to −122.3)	<0.01
5 min	16.19	0.21	0.10	0.21	0 (42.1 to 98.9)	<0.01	0.09 (−578.3 to −185.4)	<0.01
15 min	13.60	0.17	0.05	0.18	0 (103.4 to 235.6)	<0.01	0.05 (−717.4 to −257.5)	<0.01
30 min	11.59	0.15	0.04	0.13	0 (150 to 330.1)	<0.01	0.03 (−781 to −304.7)	<0.01
1 h	9.84	0.13	0.01	0.11	0 (195 to 421)	<0.01	0.02 (−810.3 to −340.6)	<0.01
EHR-Derived	30.47	0.39	0.25	0.50	0.17 (−433 to −98.5)	<0.01	Ref	Ref

## Data Availability

Code for resampling, filtering, event-window detection, and burden calculations is openly available at https://github.com/s-ranveer/ecmo_hypo_hypertension (accessed on 14 January 2026). The datasets generated and/or analyzed during the current study are available from the corresponding author on reasonable request.

## Abbreviations

EHRs: Electronic Health Records
ECMO: Extracorporeal Membrane Oxygenation
MAP: Mean Arterial Pressure
