# Supplementary material for: EHR Sampling Interval Bias Detection and Burden of Blood Pressure Excursions: Implications for Clinical Decision Support and Model Validity in Pediatric ECMO
Source: Information (Basel). Author manuscript; Available in PMC 2026 Apr 25. (PMC13108679; doi:10.3390/info17020135)
Supplement: information-17-00135-s001 [file NIHMS2167121-supplement-information-17-00135-s001.zip › information-4000023-supplementary.pdf]

Supplementary File S1

Inclusion & Exclusion Criteria

Inclusion criteria:

1. 0 – 18 years of age
2. Intra or post ECMO (< 30 days) CT or MRI
3. With or without previous cardiac arrest (e.g. eCPR)

Exclusion criteria:

1. Cyanotic congenital heart disease
2. ECMO duration < 48 hours
3. Severe cognitive deficit
4. Known pre-ECMO significant neurological injury

Median Hypotension-Hypertension Events (5-95th Percentile)

MAP Sample Frequency	Total events	Mean per Patient	Median per Patient	*IQR*
Hypotension				
5 seconds	2,358	30.23	3.00	18.00
30 seconds	2,689	34.47	4.50	18.50
1 minutes	2,928	37.54	5.00	20.75
5 minutes	1,956	25.08	4.00	14.25
15 minutes	596	7.64	1.00	5.00
30 minutes	274	3.51	0.00	2.00
1 hour	131	1.68	0.00	1.00
EHR Derived	1,766	22.64	12.00	20.25
Hypertension				
5 seconds	175	2.24	0.00	1.00
30 seconds	209	2.68	0.00	1.00
1 minutes	244	3.13	0.00	1.75
5 minutes	161	2.06	0.00	1.00
15 minutes	35	0.45	0.00	0.00
30 minutes	11	0.14	0.00	0.00
1 hour	4	0.05	0.00	0.00
EHR Derived	693	8.88	3.50	7.50

Supplemental Table S1. Summary Statistics about hypotension and hypertension **events** in 78 patients on ECMO, total events is the sum across for all patients calculated for each patient using the median blood pressure (*i.e.,* **median MAP**) measurement across different interval sizes, from every 5 seconds to every hour. For each such frequency, the columns represent the total number of events aggregated over all patients and the mean, the median, and the interquartile range of the number of events in the cohort. Each hypotension (or hypertension) event is defined as a 3-minute period where the blood pressure falls below the **5th percentile** (or exceeds the **95th percentile**) value for the patient's age group.

Median Hypotension-Hypertension Events (25-75th Percentile)

MAP Sample Frequency	Total events	Mean per Patient	Median per Patient	*IQR*
Hypotension				
5 seconds	32,444	415.95	218.00	348.75
30 seconds	36,801	471.81	258.00	410.25
1 minutes	39,817	510.47	284.50	461.50
5 minutes	26,610	341.15	192.50	305.00
15 minutes	8,675	111.22	61.50	102.00
30 minutes	4,184	53.64	28.50	48.50
1 hour	2,024	25.95	12.50	24.00
EHR Derived	8,488	108.82	75.00	89.00
Hypertension				
5 seconds	5,771	73.99	31.50	84.00
30 seconds	7,395	94.81	47.50	90.75
1 minutes	8,128	104.21	53.50	113.50
5 minutes	5,487	70.35	39.00	83.25
15 minutes	1,618	20.74	10.50	24.00
30 minutes	720	9.23	4.50	11.50
1 hour	310	3.97	1.00	5.75
EHR Derived	4,355	55.83	43.50	50.75

Supplemental Table S2. Summary Statistics about hypotension and hypertension **events** in 78 patients on ECMO, total events is the sum across for all patients calculated for each patient using the median blood pressure (*i.e.,* **median MAP**) measurement across different interval sizes, from every 5 seconds to every hour. For each such frequency, the columns represent the total number of events aggregated over all patients and the mean, the median, and the interquartile range of the number of events in the cohort. Each hypotension (or hypertension) event is defined as a 3-minute period where the blood pressure falls below the **25th percentile** (or exceeds the **75th percentile**) value for the patient's age group.

Median Hypotension-Hypertension Burden (5-95th Percentile)

MAP Sample Frequency	Total Burden	Mean per Patient	Median per Patient	*IQR*
Hypotension				
5 seconds	15.19	0.19	0.08	0.17
30 seconds	15.06	0.19	0.08	0.16
1 minute	15.00	0.19	0.08	0.16
5 minutes	14.48	0.19	0.07	0.16
15 minutes	13.29	0.17	0.06	0.16
30 minutes	12.23	0.16	0.05	0.14
1 hour	10.97	0.14	0.04	0.12
EHR Derived	15.97	0.20	0.09	0.19
Hypertension				
5 seconds	6.35	0.08	0.03	0.07
30 seconds	6.12	0.08	0.02	0.07
1 minute	5.94	0.08	0.02	0.07
5 minutes	4.98	0.06	0.02	0.06
15 minutes	3.58	0.05	0.00	0.04
30 minutes	2.64	0.03	0.00	0.02
1 hour	2.03	0.03	0.00	0.01
EHR Derived	14.20	0.18	0.10	0.19

Supplemental Table S3. Summary Statistics about the hypotensive and hypertensive **burden** in 78 patients on ECMO, , total burden is the sum across all patients, while mean and median calculated for each patient using the median blood pressure (*i.e.,* **median MAP**) measurement across different interval sizes, from every 5 seconds to every hour. For each such frequency, the columns represent the total hypotensive and hypertensive burden over all patients and the mean, the median, and the interquartile range of the patient-wise area in the cohort. Hypotensive (or hypertensive) burden is defined as the area of blood pressure curve (in mmHg-seconds) where its values fall below the **5th percentile** (or exceed the **95th percentile**) value for the patient's age group, normalized by the total time spanned by the observations for that patient, normalized by the total time in seconds spanned by the observations for that patient.

Median Hypotension-Hypertension Burden (25-75th Percentile)

MAP Sample Frequency	Total Burden	Mean per Patient	Median per Patient	*IQR*
Hypotension				
5 seconds	135.82	1.74	1.34	1.71
30 seconds	135.61	1.74	1.34	1.71
1 minute	135.44	1.74	1.34	1.71
5 minutes	134.16	1.72	1.31	1.69
15 minutes	131.24	1.68	1.24	1.70
30 minutes	127.74	1.64	1.18	1.67
1 hour	122.99	1.58	1.10	1.73
EHR Derived	134.46	1.72	1.45	1.63
Hypertension				
5 seconds	76.37	0.98	0.72	0.98
30 seconds	75.35	0.97	0.71	0.98
1 minute	74.62	0.96	0.69	0.98
5 minutes	71.32	0.91	0.64	0.97
15 minutes	65.70	0.84	0.56	0.91
30 minutes	61.20	0.78	0.46	0.86
1 hour	55.97	0.72	0.38	0.77
EHR Derived	98.52	1.26	0.99	1.30

Supplemental Table S4. Summary Statistics about the hypotensive and hypertensive **burden** in 78 patients on ECMO, , total burden is the sum across all patients, while mean and median calculated for each patient using the median blood pressure (*i.e.,* **median MAP**) measurement across different interval sizes, from every 5 seconds to every hour. For each such frequency, the columns represent the total hypotensive and hypertensive burden over all patients and the mean, the median, and the interquartile range of the patient-wise area in the cohort. Hypotensive (or hypertensive) burden is defined as the area of blood pressure curve (in mmHg-seconds) where its values fall below the **25th percentile** (or exceed the **75th percentile**) value for the patient's age group, normalized by the total time in seconds spanned by the observations for that patient.

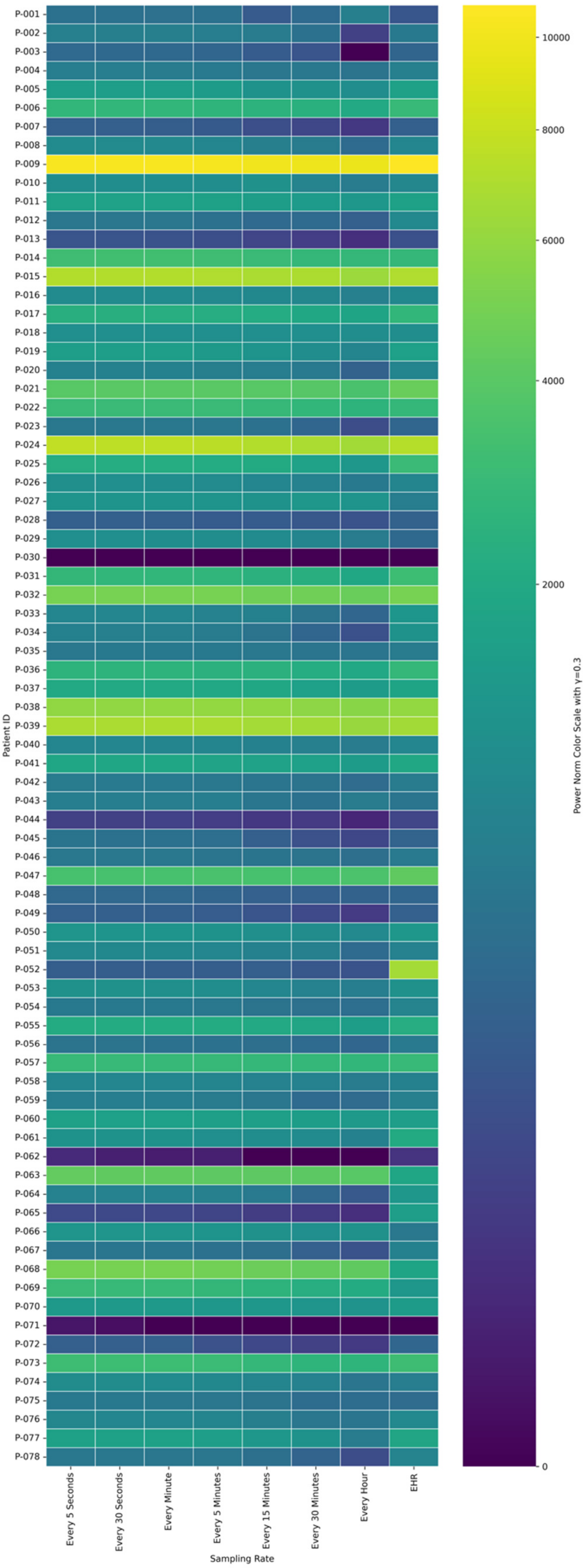
Patient ID
P-001
P-002
P-003
P-004
P-005
P-006
P-007
P-008
P-009
P-010
P-011
P-012
P-013
P-014
P-015
P-016
P-017
P-018
P-019
P-020
P-021
P-022
P-023
P-024
P-025
P-026
P-027
P-028
P-029
P-030
P-031
P-032
P-033
P-034
P-035
P-036
P-037
P-038
P-039
P-040
P-041
P-042
P-043
P-044
P-045
P-046
P-047
P-048
P-049
P-050
P-051
P-052
P-053
P-054
P-055
P-056
P-057
P-058
P-059
P-060
P-061
P-062
P-063
P-064
P-065
P-066
P-067
P-068
P-069
P-070
P-071
P-072
P-073
P-074
P-075
P-076
P-077
P-078
Every 5 Seconds
Every 30 Seconds
Every Minute
Every 5 Minutes
Every 15 Minutes
Every 30 Minutes
Every Hour
EHR
Sampling Rate
10000
8000
6000
4000
2000
0
Power Norm Color Scale with γ=0.3

Supplemental Figure S1: Heat Map comparing individual patient level burden of hypotension (10th percentile)

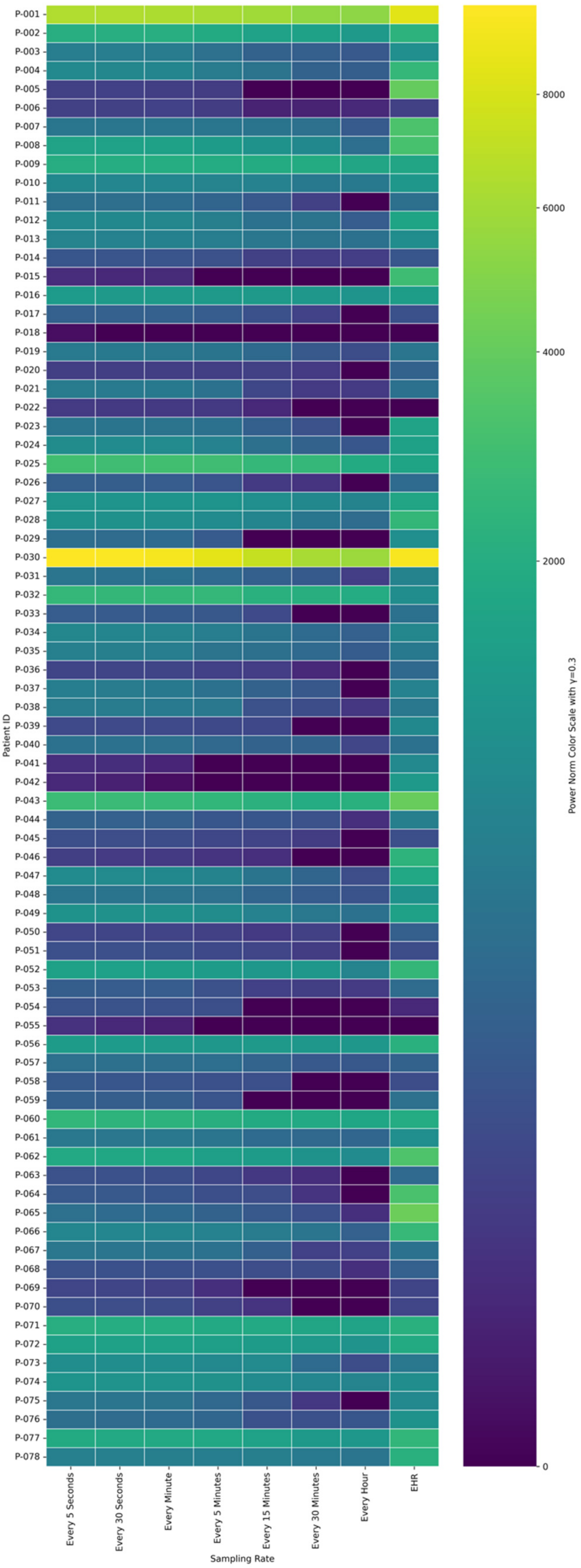

Patient ID
P-001
P-002
P-003
P-004
P-005
P-006
P-007
P-008
P-009
P-010
P-011
P-012
P-013
P-014
P-015
P-016
P-017
P-018
P-019
P-020
P-021
P-022
P-023
P-024
P-025
P-026
P-027
P-028
P-029
P-030
P-031
P-032
P-033
P-034
P-035
P-036
P-037
P-038
P-039
P-040
P-041
P-042
P-043
P-044
P-045
P-046
P-047
P-048
P-049
P-050
P-051
P-052
P-053
P-054
P-055
P-056
P-057
P-058
P-059
P-060
P-061
P-062
P-063
P-064
P-065
P-066
P-067
P-068
P-069
P-070
P-071
P-072
P-073
P-074
P-075
P-076
P-077
P-078
Every 5 Seconds
Every 30 Seconds
Every Minute
Every 5 Minutes
Every 15 Minutes
Every 30 Minutes
Every Hour
EHR
Sampling Rate
8000
6000
4000
2000
0
Power Norm Color Scale with γ=0.3

Supplemental Figure S2: Heat Map comparing individual patient level burden of hypertension (90th percentile)

Supplemental Figures

Comparing Hypotension (10 & 90th percentile) Every 5-S to EHR

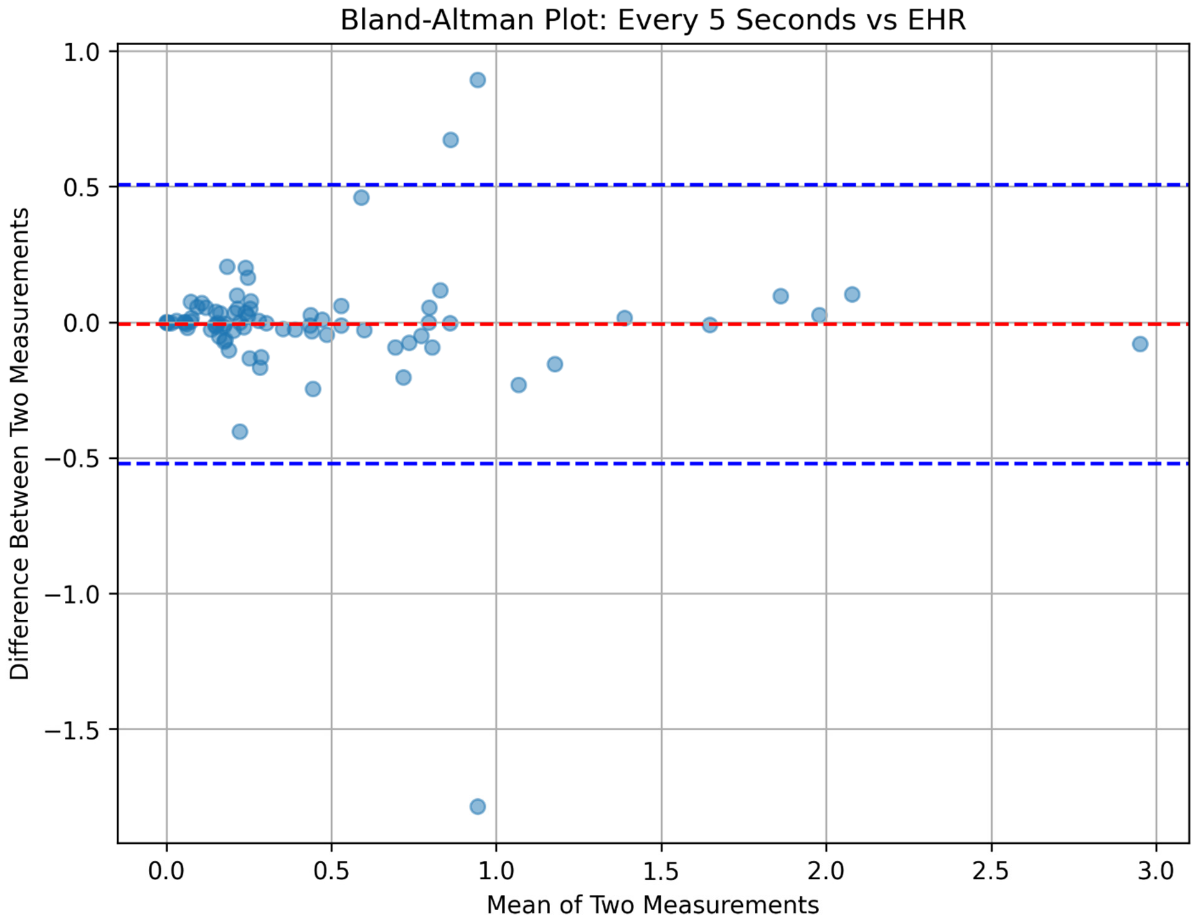

Comparing Hypotension (10 & 90th percentile) Every 5-S to 15 minutes

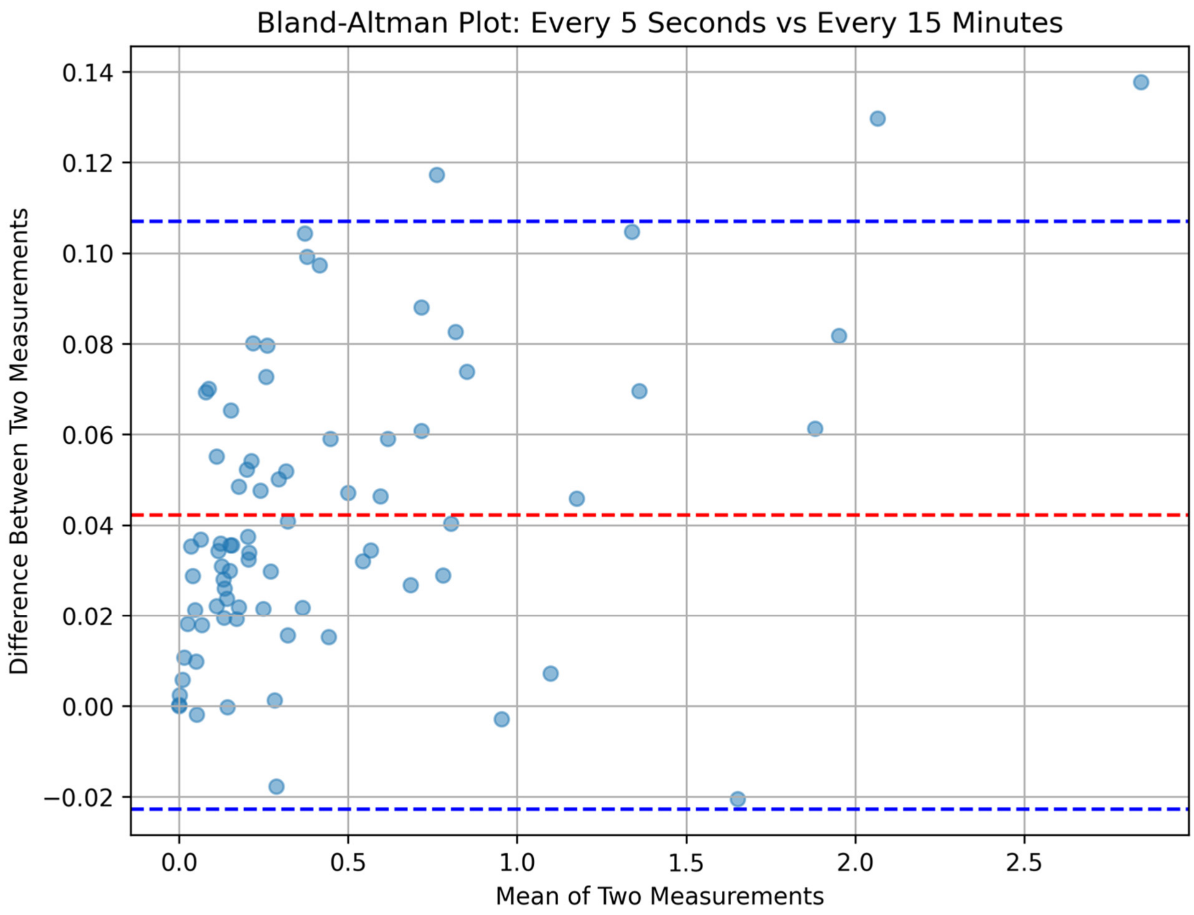

Comparing Hypotension (10 & 90th percentile) Every 5-S to 1 hour

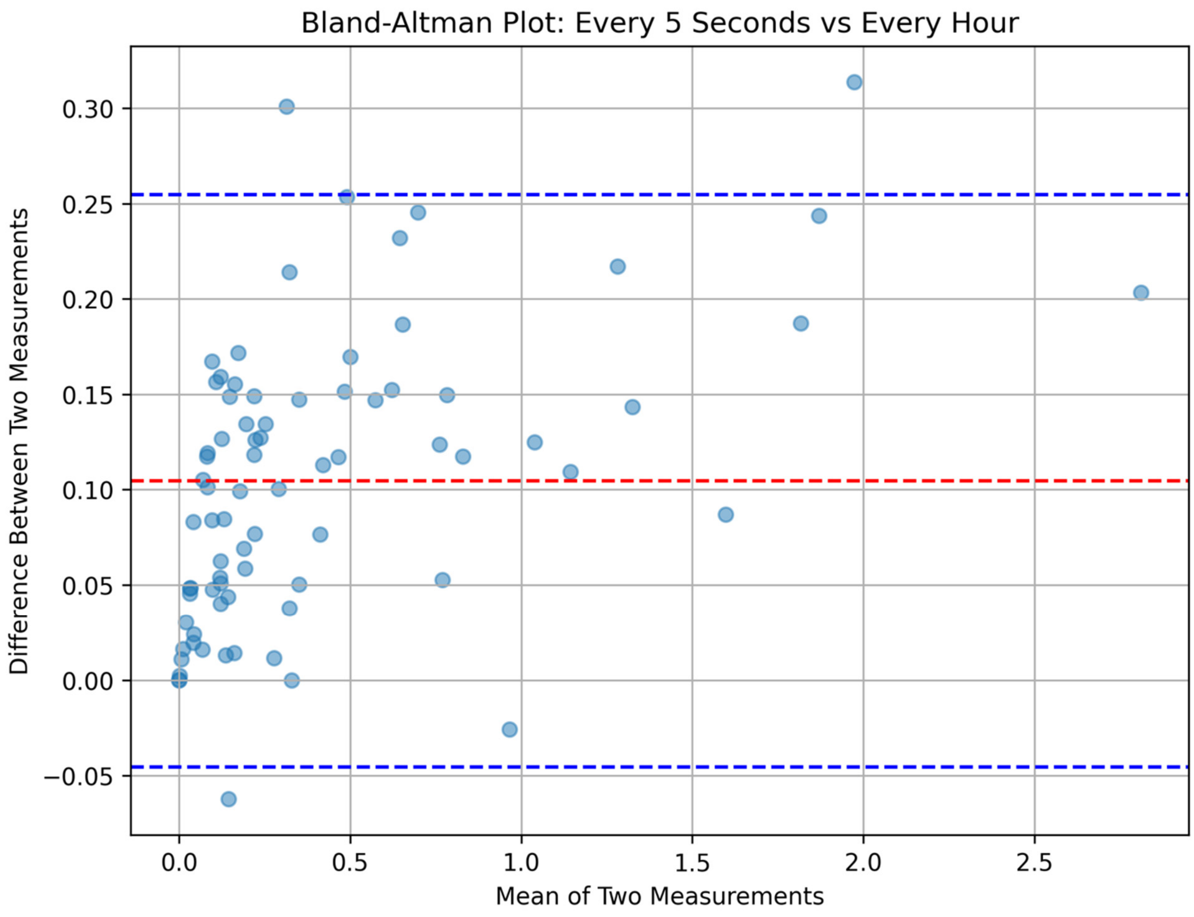

Comparing Hypertension (10 & 90th percentile) Every 5-S to EHR

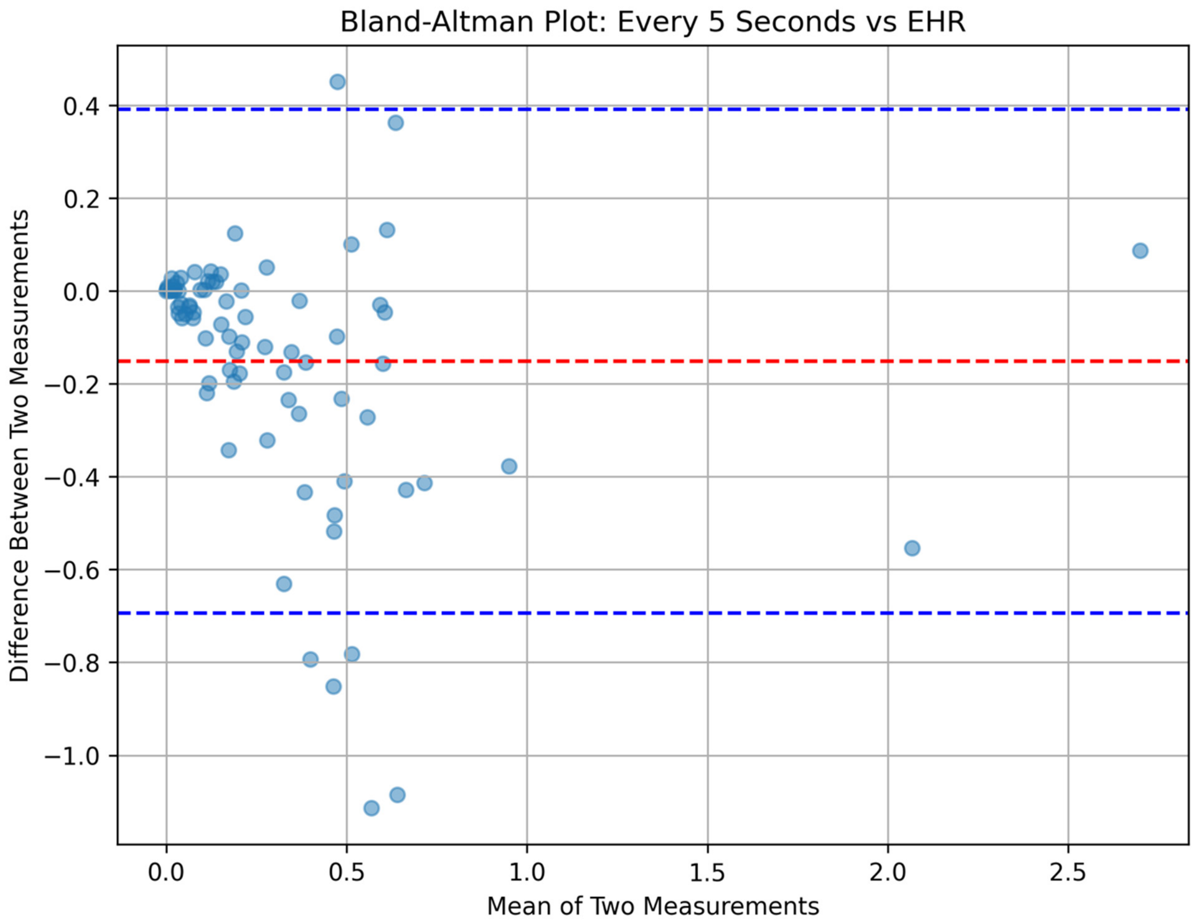

Comparing Hypertension (10 & 90th percentile) Every 5-S to 15 minutes

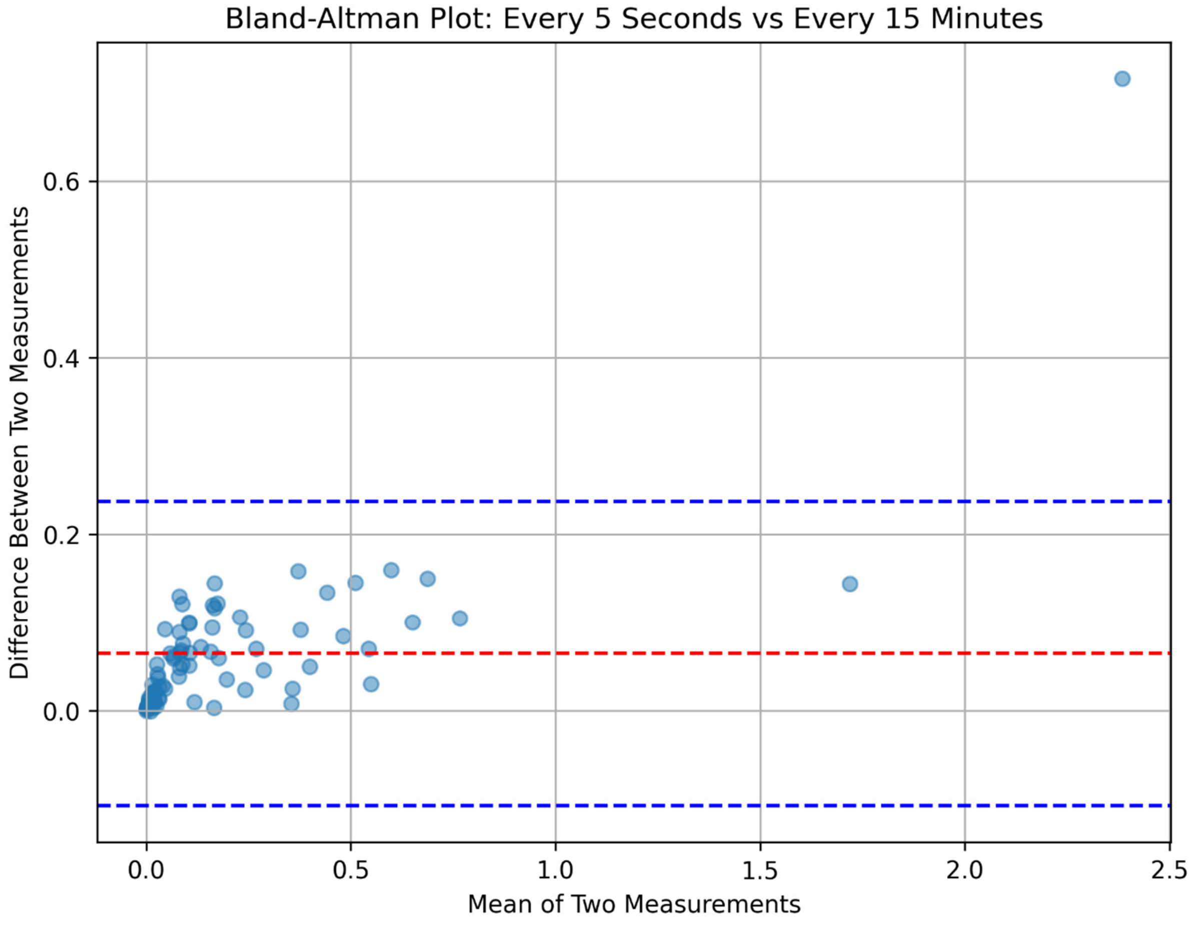

Comparing Hypertension (10 & 90th percentile) Every 5-S to 1 hour

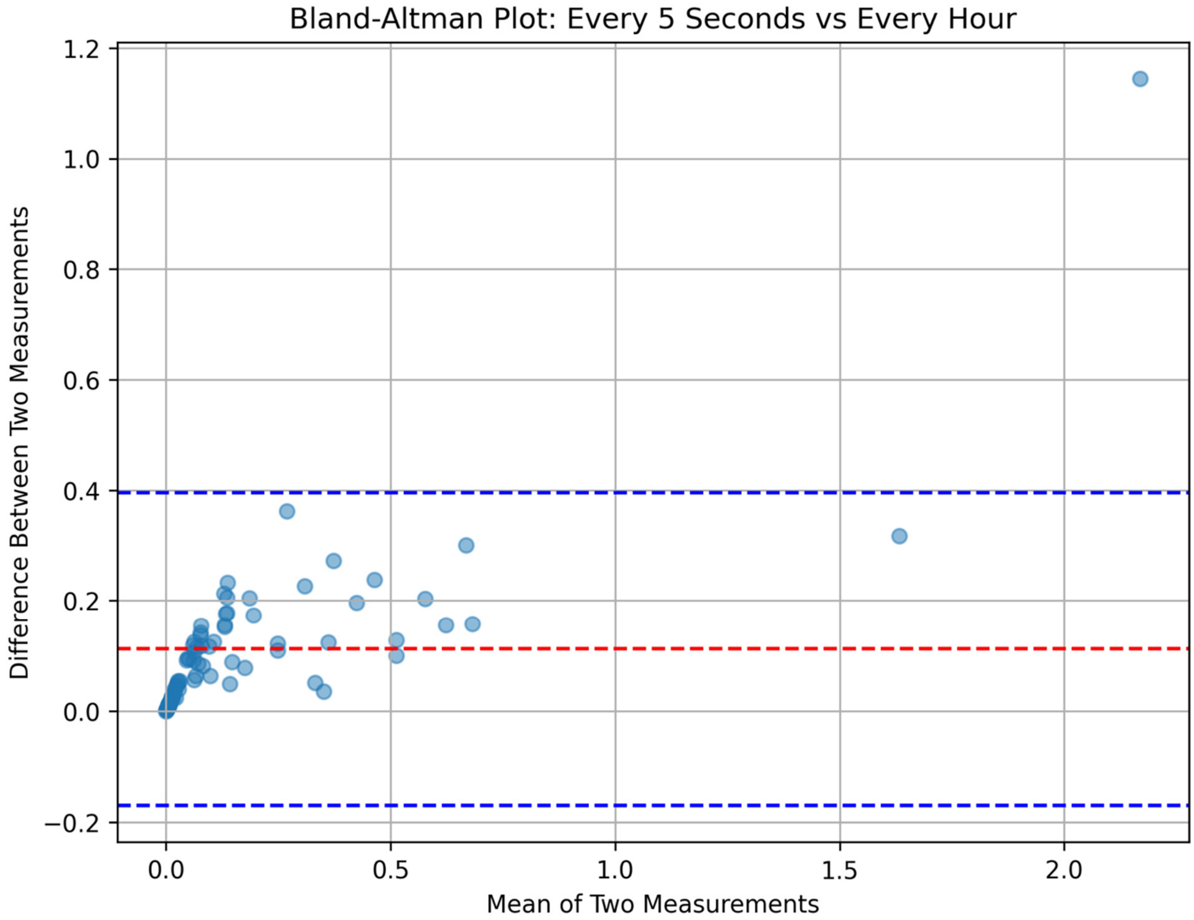
Bland-Altman Plot: Every 5 Seconds vs Every Hour
1.2
1.0
0.8
0.6
0.4
0.2
0.0
−0.2
Difference Between Two Measurements
0.0
0.5
1.0
1.5
2.0
Mean of Two Measurements

Pearson Correlation between windows and Hypotension

	Every 5 Seconds	Every 30 Seconds	Every Minute	Every 5 Minutes	Every 15 Minutes	Every 30 Minutes	Every Hour	EHR
Every 5 Seconds	1.0000	0.9993	0.9983	0.9963	0.9973	0.9976	0.9970	0.9388
Every 30 Seconds	0.9993	1.0000	0.9997	0.9985	0.9991	0.9991	0.9977	0.9432
Every Minute	0.9983	0.9997	1.0000	0.9995	0.9996	0.9992	0.9972	0.9457
Every 5 Minutes	0.9963	0.9985	0.9995	1.0000	0.9995	0.9984	0.9955	0.9505
Every 15 Minutes	0.9973	0.9991	0.9996	0.9995	1.0000	0.9992	0.9970	0.9483
Every 30 Minutes	0.9976	0.9991	0.9992	0.9984	0.9992	1.0000	0.9978	0.9453
Every Hour	0.9970	0.9977	0.9972	0.9955	0.9970	0.9978	1.0000	0.9342
EHR	0.9388	0.9432	0.9457	0.9505	0.9483	0.9453	0.9342	1.0000

Pearson Correlation between windows and Hypertension

	Every 5 Seconds	Every 30 Seconds	Every Minute	Every 5 Minutes	Every 15 Minutes	Every 30 Minutes	Every Hour	EHR
Every 5 Seconds	1.0000	0.9905	0.9799	0.9715	0.9685	0.9213	0.8620	0.7492
Every 30 Seconds	0.9905	1.0000	0.9973	0.9927	.9802070371049219	0.9201	0.8534	0.7833
Every Minute	0.9799	0.9973	1.0000	0.9981	0.9822	0.9169	0.8487	0.7894
Every 5 Minutes	0.9715	0.9927	0.9981	1.0000	0.9826	0.9180	0.8545	0.7792
Every 15 Minutes	0.9685	0.9802	0.9822	0.9826	1.0000	0.9493	0.9043	0.7452
Every 30 Minutes	0.9213	0.9201	0.9169	0.9180	0.9493	1.0000	0.9633	0.5808
Every Hour	0.8620	0.8534	0.8487	0.8545	0.9043	0.9633	1.0000	0.4845
EHR	0.7492	0.7833	0.7894	0.7792	0.7452	0.5808	0.4845	1.0000
